# Bird harvesting practices and knowledge, risk perceptions, and attitudes regarding avian influenza among Canadian First Nations subsistence hunters: implications for influenza pandemic plans

**DOI:** 10.1186/1471-2458-14-1113

**Published:** 2014-10-28

**Authors:** Nadia A Charania, Ian D Martin, Eric N Liberda, Richard Meldrum, Leonard JS Tsuji

**Affiliations:** Department of Environment and Resource Studies, University of Waterloo, 200 University Avenue West, N2L 3G1 Waterloo, Ontario Canada; Department of Physical & Environmental Sciences, University of Toronto Scarborough, 1265 Military Trail, M1C 1A4 Toronto, Ontario Canada; School of Occupational and Public Health, Ryerson University, 350 Victoria Street, POD 247H, M5B 2K3 Toronto, Ontario Canada

**Keywords:** Avian influenza, Birds, Wild game, First Nations, Canada, Subsistence hunting, Harvesting, Pandemic plans, Risk perception

## Abstract

**Background:**

There is concern of avian influenza virus (AIV) infections in humans. Subsistence hunters may be a potential risk group for AIV infections as they frequently come into close contact with wild birds and the aquatic habitats of birds while harvesting. This study aimed to examine if knowledge and risk perception of avian influenza influenced the use of protective measures and attitudes about hunting influenza-infected birds among subsistence hunters.

**Methods:**

Using a community-based participatory research approach, a cross-sectional survey was conducted with current subsistence hunters (n = 106) residing in a remote and isolated First Nations community in northern Ontario, Canada from November 10–25, 2013. Simple descriptive statistics, cross-tabulations, and analysis of variance (ANOVA) were used to examine the distributions and relationships between variables. Written responses were deductively analyzed.

**Results:**

ANOVA showed that males hunted significantly more birds per year than did females (F_1,96_ = 12.1; p = 0.001) and that those who hunted significantly more days per year did not perceive a risk of AIV infection (F_1,94_ = 4.4; p = 0.040). Hunters engaged in bird harvesting practices that could expose them to AIVs, namely by cleaning, plucking, and gutting birds and having direct contact with water. It was reported that 18 (17.0%) hunters wore gloves and 2 (1.9%) hunters wore goggles while processing birds. The majority of hunters washed their hands (n = 105; 99.1%) and sanitized their equipment (n = 69; 65.1%) after processing birds. More than half of the participants reported being aware of avian influenza, while almost one third perceived a risk of AIV infection while harvesting birds. Participants aware of avian influenza were more likely to perceive a risk of AIV infection while harvesting birds. Our results suggest that knowledge positively influenced the use of a recommended protective measure. Regarding attitudes, the frequency of participants who would cease harvesting birds was highest if avian influenza was detected in regional birds (n = 55; 51.9%).

**Conclusions:**

Our study indicated a need for more education about avian influenza and precautionary behaviours that are culturally-appropriate. First Nations subsistence hunters should be considered an avian influenza risk group and have associated special considerations included in future influenza pandemic plans.

## Background

Influenza A viruses may cause pandemics at unpredictable, irregular intervals resulting in devastating social and economic effects worldwide [1]. Wild aquatic birds in the orders Anseriformes and Charadriiformes are the natural hosts for influenza A viruses; these viruses have generally remained in evolutionary stasis and are usually non-pathogenic in wild birds [2, 3]. Most avian influenza viruses (AIVs) primarily replicate in the intestinal tract of wild birds and are spread amongst birds via an indirect fecal-oral route involving contaminated aquatic habitats [4]. Humans who are directly exposed to the tissues, secretions, and excretions of infected birds or water contaminated with bird feces can become infected themselves [2, 4, 5]. The transmission of an AIV from a bird to a human has significant pandemic potential as it may result in the direct introduction of a novel virus strain or allow for the creation of a novel virus strain via reassortment [3, 5].

The transmission of AIVs from birds to humans depends on many factors, such as the susceptibility of humans to the virus and the frequency and type of contact [2, 5]. Most AIVs are generally inefficient in infecting humans; however, there have been documented cases of AIVs transmitting directly from infected birds to humans [6, 7]. During the 1997 Hong Kong “bird flu” incident, there was demonstrated transmission of highly pathogenic avian influenza (HPAI) A virus (H5N1) from infected domesticated chickens to humans [3]. More recently, some Asian countries have reported human infections of avian influenza A virus (H7N9) with most patients having a history of exposure to live poultry in wet markets [8]. As such, most pandemic plans include special considerations (e.g., enhanced surveillance, prioritization for vaccination, and antiviral prophylaxis) for avian influenza risk groups that include humans who come in close, frequent contact with domestic birds, such as farmers, poultry farm workers, veterinarians, and livestock workers [9, 10].

Longitudinally migrating wild birds appear to play a primary role in influenza transmission and there is increased concern about the introduction of HPAI virus strains in North America from Eurasia, as migratory flyways around the world intersect [3, 4]. Thus, bird hunters may also be at risk as hunting and processing practices directly expose them to the bodily fluids of wild birds and water potentially contaminated with bird feces [5, 11]. Although the risk of AIV infection while hunting and processing wild birds is assumed to be very low [5], transmission has been previously reported. One study reported serologic evidence of past AIV infection in a recreational duck hunter and two wildlife professionals, inferring direct transmission of AIVs from wild birds to humans [12]. Another study reported that recreational waterfowl hunters were eight times more likely to be exposed to avian influenza-infected wildlife compared to occupationally-exposed people and the general public [13]. A study conducted in rural Iowa, USA, reported that participants who hunted wild birds had increased antibody titers against avian H7 influenza virus [14]. Further, in the Republic of Azerbaijan, HPAI H5N1 infection in humans is suspected to be linked to defeathering infected wild swans (*Cygnus*) [15].

Since handling wild birds and having contact with the aquatic habitats of wild birds are potential transmission pathways for AIV infections in hunters, it is important to better understand hunters’ knowledge and risk perceptions of avian influenza and include special considerations in pandemic plans. This is particularly important for some Canadian Aboriginal (First Nations, Inuit, and Métis) populations whose hunting of wild birds represents subsistence harvesting as opposed to a recreational activity [16]. Herein, subsistence harvesting will refer collectively to activities associated with hunting, fishing, trapping, and gathering of animals and other food for personal, family, and community consumption [17, 18]. The practice of subsistence harvesting for some Canadian Aboriginal populations, such as the Cree First Nations of the Mushkegowuk region, is culturally and economically important with the majority of hunters harvesting wild birds [17, 19]. Traditional land-based harvesting activities are economically valuable for the region and can reduce external economic dependence [17]. Moreover, as there are many physical, nutritional, and social benefits of this practice, it is a vital, well-established component of health and well-being in Canadian Aboriginal communities [20]. For instance, as Canadian Aboriginal populations, particularly those residing in geographically remote and isolated communities, experience a high prevalence of household food insecurity [21, 22], subsistence harvesting can provide an important source of healthy traditional foods and lessen the reliance on costly market foods.

The potential of AIV infection while hunting and harvesting wild birds varies with geographical areas, seasons, and specific activities [5, 11, 12]. Moreover, previous studies have shown that knowledge and risk perception of avian influenza can positively influence compliance with recommended protective health behaviours [23, 24]. We conducted a cross-sectional survey of the bird harvesting practices and knowledge, risk perceptions, and attitudes regarding avian influenza among Canadian First Nations subsistence hunters. The purpose of this study was to examine if knowledge and risk perception of avian influenza influenced the use of personal protection measures and attitudes about hunting influenza-infected birds. The implications for addressing the special considerations of Canadian First Nations subsistence hunters in pandemic plans will be discussed.

## Methods

### Community-based participatory research approach

The present study employed a community-based participatory research (CBPR) approach since the hallmark principles of CBPR can foster the engagement of Aboriginal populations and participatory methods have previously been a successful approach to partnering with Aboriginal communities [25–27]. As such, the research topic was locally relevant as it stemmed from previous research conducted in the region that explored culturally-appropriate measures to mitigate the effects of an influenza pandemic in the setting of a remote and isolated Canadian First Nations community [28]. Residents of the study community expressed questions and concerns about the transmission potential of AIVs from influenza-infected wild birds to subsistence hunters. Thus, the present study was specifically developed and conducted to address the identified questions and concerns.

Following a CBPR approach, collaboration occurred throughout the research process between the researchers and a community-based advisory group (CBAG) comprised of two community representatives from the study community [29–31]. The two members of the CBAG were of First Nations heritage and were particularly interested in the topic at hand and desired to be involved. The CBAG helped design the study and was part of the iterative process of developing the survey questions and layout. The CBAG also provided input during the data analysis process, on the interpretation of results, and aided with disseminating the results to the community. CBPR endeavors aim to use the knowledge generated to achieve action-oriented outcomes for the involved community [29, 32]. At the request of the CBAG, the results of this study were disseminated via an oral presentation to community members during a lunch-and-learn activity in June 2014. An information sheet explaining avian influenza and recommended precautionary behaviours created by Health Canada was distributed to attendees [33]. Information about emerging avian influenzas that currently are of pandemic concern and the information sheet were also incorporated into the community’s influenza pandemic plan as a newly created appendix section.

Approval to conduct this research was granted by the Office of Research Ethics at the University of Waterloo (ORE #16534), and was supported by the Band Council (locally elected First Nations government body) of the involved community.

### Study area, population, and data collection

The study community (name omitted for anonymity purposes) is considered remote (i.e., nearest service center with year-round road access is located over 350 kilometers away) and isolated (i.e., accessible only by airplanes year-round) [10]. The Cree First Nations community belongs to the Mushkegowuk region which is located in northern Ontario, Canada along the western shores of James Bay and the southern portion of Hudson Bay [17, 19]. The region is a productive wildlife area and the majority of hunters partake in the spring and fall bird harvests [34].

The cross-sectional survey was conducted in English (as suggested by the CBAG) from November 10–25, 2013. The time period was chosen to maximize participation, as most hunters would have returned from fall hunting activities. The survey was based on previous literature [11] and was developed in collaboration with the CBAG to ensure that it adequately addressed the objectives of the study and was culturally-appropriate. The survey employed closed-ended questions to gain a better understanding of First Nations hunters’ general harvesting practices, knowledge and risk perception of avian influenza, and attitudes about hunting influenza-infected birds. Open-ended questions were also included to allow for participants to describe their risk perceptions of AIV infection while harvesting birds as well as any additional concerns. Basic demographic questions to record the age and sex of participants were also included.

Community First Nations subsistence hunters were invited to participate by the lead author (NAC) and a local community research assistant during individual meetings. The research assistant was of First Nations descent and a prominent Elder in the community. Being fluent in the Cree language, the assistant acted as a Cree translator upon request by the survey respondents. A current community housing list (updated in November 2013) which recorded all known community members living in First Nations (Band) households was used by the research assistant to identify eligible participants. Contemporary harvesting practices in the region typically involve multiple short trips versus traditional long trips [34]. To include as many hunters as possible from the study community, eligible participants were defined as current hunters, a group which included “intensive”, “active”, and “occasional” hunters (for definitions, see [17]). In addition to being a current hunter, participants were required to be First Nations (Band member), an adult (18 years old and over), and available to complete the survey in person during the study period to be eligible. Both male and female hunters were approached as it is widely recognized in Cree First Nations that both sexes play an important role while subsistence harvesting [35].

When approached, the participants were provided with an information/recruitment letter and the study was explained in English or Cree as required. Informed verbal consent was obtained, being culturally appropriate for the region [31, 36]. Incentives were not offered for participation. As participants preferred to complete the survey alone on their own time, a convenient time and location was arranged to collect the completed survey. Up to five follow-up visits and new survey copies were provided if the survey was not completed at the specified time and if the person was still interested in participating.

### Data management and analyses

Collected surveys were coded by an identification number to maintain confidentiality of the participants. The CBAG was consulted to determine how to code inexact responses. Of note, it was decided that if a participant responded with a range of numbers, the median value was recorded. If a participant selected all of the possible response options or only provided a written response, the result was recorded as missing data. In instances where a pattern was observed amongst participants’ written responses, the responses were coded according to newly created response options approved by the CBAG to maintain the integrity of the data.

Sample size for individual statistical analyses varied from 88 to 106, as not all participants answered each survey question; thus, presented percentages may not always equal 100% owing to missing data. Simple descriptive statistics were used to examine the distributions of variables pertaining to general harvesting practices, knowledge and risk perception of avian influenza, and attitudes about hunting influenza-infected birds. Cross-tabulations, as 2 × 2 contingency analyses, were used to examine the relationships between each of the main effects of sex, awareness of avian influenza, and risk perception of AIV infection by precautionary behaviours and attitudes about hunting influenza-infected birds. In instances where the expected cell count was less than five, the Fisher’s Exact Test was used in preference to the Pearson chi-square test. Absolute values greater than 1.96 of the adjusted standard residual (ASR) indicated a significant departure from the expected count and therefore considered to be a major contributor to the observed chi-square result.

The influence of outlier values for continuous dependent variables (age, years of hunting, days of hunting per year, birds hunted per year) was examined using boxplots of raw and log transformed data. Owing to the presence of outlier values, we log-transformed values for days of hunting and number of birds hunted per year to satisfy the homogeneity of variance assumption of analysis of variance (ANOVA). It was decided that one individual’s improbable response for number of birds hunted per year should be removed as it continued to distort the results. Also, one individual’s response for years of hunting was recorded as missing data since the response did not reflect the age of the participant. Differences in mean values of these dependent variables between groups for sex, awareness of avian influenza, and risk perception of AIV infection were examined using ANOVA. Statistical results were considered to be significant at p < 0.05. Data analyses were carried out using SPSS version 22 (SPSS Inc., Chicago, Illinois, U.S.A).

Written responses to the two open-ended questions and any additional comments were manually transcribed verbatim into electronic format to facilitate organization and coding. Qualitative coding of the transcribed data was conducted using QSR NVivo® version 9.2 (QSR International Pty Ltd., Doncaster, Victoria, Australia). Responses were deductively analyzed following a template organizing approach using the survey questions as a coding template [37, 38]. Analyzing the data was an iterative process conducted multiple times by the lead author (NAC) and findings were presented to the CBAG as a way of member checking to verify the results [37].

## Results

A total of 173 participants in the censused community were deemed eligible to participate given the inclusion criteria and of these, 126 received surveys, for a 73% contact rate. Of the 126 distributed surveys, 106 completed surveys were returned, representing an 84% cooperation rate. Overall, a response rate of 61% was achieved. Of the 106 community members that participated in the survey, 80 (75.5%) were male and 26 (24.5%) were female. The untransformed demographic and harvesting characteristics of the participants are presented in Table 1.

**Table 1:** **Demographic and harvesting characteristics of Canadian First Nations subsistence hunters residing in the study community (n = 106), November 10–25, 2013**

	n	Minimum	Maximum	Mean	Std. deviation
**Demographic information**					
Age	92	18	76	43.3	12.9
**Harvesting characteristics**					
Years of hunting	99	1	65	27.2	14.0
Days of hunting per year	105	1	200	26.2	30.5
Number of birds hunted per year	100	0	200	42.6	40.6

All who responded participated in the spring/summer hunting activities (n = 105; 99.1%) with fewer hunters participating during the fall (n = 57; 53.8%) and winter (n = 16; 15.1%) seasons. During these hunts, 98.1% of participants hunted Canada geese (*Branta canadensis*), 88.7% hunted various species of ducks (*Anatinae*), 69.8% hunted lesser snow geese (*Anser c. caerulescens*, also referred to as wavies), and 43.4% hunted species of shorebirds (*Charadriiformes*).

**Figure 1:**
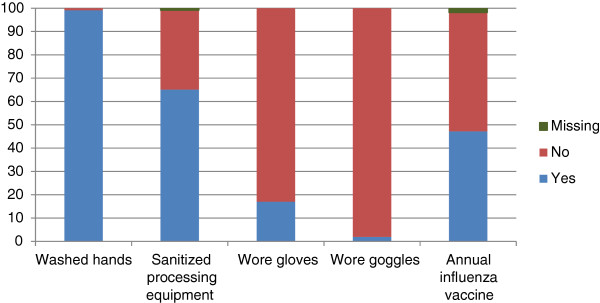
**Compliance with recommended protective health measures among Canadian First Nations subsistence hunters residing in the study community (n = 106), November 10–25, 2013.**

While hunting, the majority of participants reported having direct contact with water (n = 89; 84.0%). Bird harvesting practices were generally similar whether camping in the bush or at home; thus, only results pertaining to camping in the bush are presented. In the bush, most hunters processed the birds themselves (n = 72; 67.9%) or a family member was involved (n = 67; 63.2%). Most hunters partook in all of the bird processing activities in the bush; the percentage of participants who reported cleaning, plucking, and gutting the birds were 74.5%, 94.3%, and 77.4% respectively. Regarding the use of precautionary measures while processing birds in the bush, it was reported that 18 (17.0%) hunters wore gloves and 2 (1.9%) hunters wore goggles. In the bush, the majority of hunters washed their hands (n = 105; 99.1%) and sanitized their equipment (n = 69; 65.1%) after processing birds. Moreover, about half of the participants (n = 50; 47.2%) reported receiving the annual vaccination against seasonal human influenza viruses (Figure 1).

The total frequency and percentage of participants’ knowledge of avian influenza, risk perception of AIV infection, and attitudes about hunting influenza-infected birds are presented in Table 2. Approximately half of the participants (n = 56; 52.8%) reported being generally aware of avian influenza, but few were aware of the signs and symptoms of avian influenza in birds (n = 16; 15.1%) or humans (n = 9; 8.5%).

**Table 2:** **Frequency and percentage** ^**a**^ **of knowledge of avian influenza, risk perception of avian influenza virus infection, and attitudes about hunting influenza-infected birds among Canadian First Nations subsistence hunters residing in the study community (n = 106), November 10–25, 2013**

	All hunters		Males		Females	
	No (%)	Yes (%)	No (%)	Yes (%)	No (%)	Yes (%)
**Knowledge**						
Aware of avian influenza	49 (46.2)	56 (52.8)	37 (46.3)	42 (52.5)	12 (46.2)	14 (53.8)
Aware of signs and symptoms of avian influenza in birds	89 (84.0)	16 (15.1)	67 (83.8)	12 (15.0)	22 (84.6)	4 (15.4)
Aware of signs and symptoms of avian influenza in humans	95 (89.6)	9 (8.5)	74 (92.5)	4 (5.0)	21 (80.8)	5 (19.2)
**Risk perception**						
Perceived risk of avian influenza virus infection	68 (64.2)	29 (27.4)	52 (65.0)	23 (28.8)	16 (61.5)	6 (23.1)
**Attitudes**						
Cease hunting if avian influenza detected in North American birds	60 (56.6)	43 (40.6)	49 (61.3)	29 (36.3)	11 (42.3)	14 (53.8)
Cease hunting if avian influenza detected in Province of Ontario birds	54 (50.9)	45 (42.5)	45 (56.3)	30 (37.5)	9 (34.6)	15 (57.7)
Cease hunting if avian influenza detected in Regional birds	46 (43.4)	55 (51.9)	39 (48.8)	37 (46.3)	7 (26.9)	18 (69.2)

^a^Percentages may not always equal 100% owing to missing data.

Some participants (n = 29; 27.4%) perceived a risk of contracting avian influenza while harvesting birds.

“*Just wondering every time we go out hunting geese in the spring, if any of the geese that come in [the] spring are carrying the flu*” (Participant #41).

“*Yes there is a risk [be]cause the birds [are] from the South … who knows what they’ll catch out there*” (Participant #103).

“*It will concern me if the bird flu is here on our Land and I wouldn’t be sure about hunting birds*” (Participant #42).

On the other hand, many participants did not perceive a risk of AIV infection while harvesting birds, since local regional birds were not perceived to be infected with avian influenza.

“*I thought there was only bird flu in Asia …*” (Participant #24).

“*If birds were sick, I don’t think they would make it this far [North]*” (Participant #70).

“*No reports that bird flu has arrived in this area and people are not getting sick*” (Participant #36).

Detection of avian influenza in wild birds in nearby geographic areas would reportedly influence the participants’ harvesting behaviour. The frequency of participants who would cease harvesting birds was highest if avian influenza was detected in local regional birds (n = 55; 51.9%). It was reported that 45 (42.5%) respondents would stop hunting if avian influenza was found in birds from within the Province of Ontario, and 43 (40.6%) respondents would stop hunting if the virus was found in North American birds. For all of the aforementioned scenarios, some participants added written responses indicating that they were not sure if they would stop hunting and requested relevant information. The majority of respondents also were interested in receiving information about avian influenza transmission (n = 83; 78.3%), flyways of migrating birds (n = 79; 74.5%), and precautions to minimize exposure (n = 82; 77.4%).

**Figure 2:**
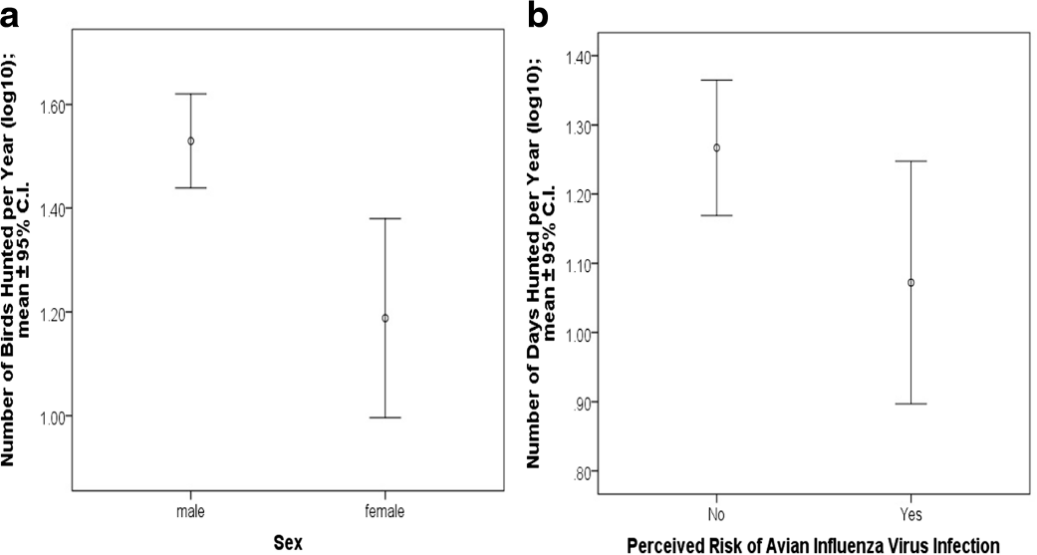
**Analysis of variance for number of birds hunted per year by males and females (a) and number of days hunted per year by perceived risk of avian influenza virus infection while harvesting birds (b) among Canadian First Nations subsistence hunters residing in the study community (n = 106), November 10–25, 2013.**

ANOVA showed that males hunted significantly more birds per year than did females (F_1,96_ = 12.1; p = 0.001; Figure 2). No significant difference in mean values of age, years of hunting, and days of hunting per year was observed between males and females. ANOVA did not identify any significant differences in mean values of age, years of hunting, days of hunting per year, and number of birds hunted per year between those who were or were not aware of avian influenza. However, ANOVA did show that those who hunted significantly more days per year did not perceive a risk of AIV infection while harvesting birds (F_1,94_ = 4.4; p = 0.040; Figure 2). No significant difference in mean values of age, years of hunting, and number of birds hunted per year was observed between those who did or did not perceive a risk of AIV infection.

For all participants, in 2 × 2 contingency analysis, a significant dependence was observed between awareness of avian influenza and risk perception of AIV infection (Pearson χ^2^ = 4.456; p = 0.035) (Table 3). An ASR of +2.1 indicated that participants aware of avian influenza were significantly more likely to perceive a risk of AIV infection while harvesting birds. No significant dependence was seen between sex and awareness of avian influenza or sex and perceived risk of AIV infection.

**Table 3:** **Cross-tabulation for awareness of avian influenza by risk perception of avian influenza infection while harvesting birds among Canadian First Nations subsistence hunters residing in the study community (n = 106), November 10–25, 2013**

			Perceived risk of avian influenza infection while harvesting birds		Total
			No	Yes	
Aware of avian influenza	No	Count	37	9	46
		Adjusted Residual	+2.1	-2.1	
	Yes	Count	31	20	51
		Adjusted Residual	-2.1	+2.1	

A significant dependence was observed between sex and the attitude of ceasing hunting if influenza was detected in regional birds (Pearson χ^2^ = 4.123; p = 0.042) (Table 4). An ASR of -2.0 indicted that males were significantly less likely to stop hunting if influenza was detected in the local regional birds. No significant dependence was observed between the two main effects of awareness of avian influenza and perceived risk of AIV infection by attitudes about hunting influenza-infected birds.

**Table 4:** **Cross-tabulation for sex by cease hunting if influenza detected in Regional birds among Canadian First Nations subsistence hunters residing in the study community (n = 106), November 10–25, 2013**

			Cease hunting if influenza detected in Regional birds		Total
			No	Yes	
Sex	Male	Count	39	37	76
		Adjusted Residual	+2.0	-2.0	
	Female	Count	7	18	25
		Adjusted Residual	-2.0	+2.0	

A significant dependence also was observed between awareness of avian influenza and the precautionary behaviour of sanitizing equipment after processing birds while camping in the bush (Pearson χ^2^ = 4.070; p = 0.044) (Table 5). An ASR of +2.0 indicated that a significantly greater frequency of aware participants were among those who cleaned their bird processing equipment. No significant dependence was observed between awareness of avian influenza by any of the other recommended precautions to be used while harvesting birds. Moreover, no significant dependence was observed between the two main effects of sex and perceived risk of AIV infection by any of the precautionary behaviours.

**Table 5:** **Cross-tabulation for awareness of avian influenza by sanitizing bird processing equipment in the bush among Canadian First Nations subsistence hunters residing in the study community (n = 106), November 10–25, 2013**

			Sanitize bird processing equipment in the bush		Total
			No	Yes	
Aware of avian influenza	No	Count	21	27	48
		Adjusted Residual	+2.0	-2.0	
	Yes	Count	14	42	56
		Adjusted Residual	-2.0	+2.0	

## Discussion

### Harvesting activities

As mentioned, the potential of AIV infection while hunting and processing wild birds varies with specific practices, seasons, and geographical areas [5, 11, 12]. The hunters reported being in frequent contact with wild birds, as some participants hunted for more than 100 days per year and harvested up to 200 birds per year. Our findings indicated that First Nations subsistence hunters were involved in bird harvesting practices, such as processing the birds and having direct contact with water in the bush, that pose an increased hazard to AIV infections among this subpopulation. The main proposed pathway of transmission of AIV to humans is close contact between the tissues, secretions, and excretions of an infected bird and the respiratory tract, gastrointestinal tract, or conjunctiva of a human [2, 7, 39]. Infected birds shed copious amounts of virus particles in their feces which can also contaminate the environment and bodies of water [40, 41]. Our findings revealed that the majority of hunters had direct contact with water and cleaned, plucked, and gutted the wild birds themselves. If processing an influenza-infected wild bird in this manner, hunters may be exposed to virus-laden tissues, secretions, and excretions [2, 5]. The use of personal protective equipment was not routine practice as most hunters did not wear gloves and goggles to protect themselves while processing birds. However, most hunters reported using other measures of personal protection, such as washing their hands and cleaning their equipment, which can limit post-harvest AIV exposure.

The timing of the hunters’ bird harvesting activities in relation to when the prevalence peaks for AIVs and human influenza viruses is of particular interest. Similar to previous reports, our study revealed that the majority of hunters were involved in the spring and fall bird harvests [16, 19, 34]. The timing of these harvests is in relation to freeze-up and break-up events in the region which varies every year, but generally runs from April to October [42]. During these harvests, participants reported hunting migratory wild birds that are potential carriers of AIVs as all known influenza A virus subtypes have been identified in these birds [3, 43]. For instance, in North American wild ducks, AIV prevalence peaks around late summer/early fall prior to south bound migration, with highest virus isolation rates reported in juvenile ducks [44, 45]. On the other hand, previous studies have reported relatively low prevalence of AIVs in Canada geese regardless of the season [45, 46]. Moreover, in Canada, the peak season of influenza A infection in humans typically runs from November to April [33]. Similar to another study, our results suggest that the possibility of co-infection with AIVs and human influenza viruses resulting in a reassortment event is unlikely as the timing of the hunters’ potential exposure to AIVs is different from that of seasonal human influenza viruses [5].

Based on previous studies, the surveyed participants generally hunt for wild birds around the southwestern coast of Hudson Bay and the western coast of James Bay which is along the Mississippi migratory flyway [3, 34, 47, 48]. Migratory flyways around the world intersect, particularly between eastern Eurasia and Alaska and between Europe and eastern North America, raising concerns about the exchange of AIVs between the Eurasian and American virus superfamilies [3, 43]. Intercontinental exchange of entire AIV genomes has not yet been reported and Eurasian HPAI virus subtypes have not been previously detected in North American migratory birds [43, 49]. However, reassortment events between the two lineages has been reported, notably in Alaska and along the northeastern coast of Canada [43, 49–51]. These observations suggest that the introduction of a novel AIV is more likely to occur along the Pacific and Atlantic coasts of North America, but once introduced, it has been suggested that migration to major congregation sites may disperse the novel AIV across flyways [49, 51, 52].

### Awareness, risk perception, and attitudes

Approximately half of our study participants were generally aware of avian influenza (52.8%), which is lower than previous studies conducted with bird hunters in the USA (86%) and poultry workers in Nigeria (67.1%) and Italy (63.8%) [11, 23, 53]. Similar to a previous study, our findings indicated that a general awareness of avian influenza was more common among the surveyed bird hunters compared to knowledge of the signs and symptoms [11]. Previous studies conducted with high-risk populations in Thailand and Laos also reported limited knowledge of the key signs and symptoms of avian influenza [54, 55]. Almost one third of surveyed participants perceived a risk of contracting avian influenza while hunting and processing birds which is similar to the values found in other studies [24, 56].

Our results revealed that the frequency of First Nations hunters who would cease harvesting birds increased as AIV was detected in more nearby geographic areas. This observation aligns with findings from a previous study; however, the percentage of hunters who would stop was relatively higher in our study as only 3% and 19% of active duck hunters in Georgia, USA reported that they would stop hunting if HPAI were found in duck populations in USA and the state of Georgia, respectively [11]. This result is interesting as harvesting activities are integral to First Nations’ culture and an important source of healthy food, especially in communities experiencing food insecurity [17, 20, 22].

Our findings suggested that being aware of avian influenza or perceiving a risk of AIV infection did not influence the hunters’ decision to cease harvesting influenza-infected birds. However, those who were knowledgeable were more likely to clean their equipment after processing birds in the bush. This finding suggests that First Nations hunters are not only willing to use precautionary measures while harvesting birds, but that improving their knowledge level may lead to an increased use of recommended precautionary measures. Previous studies also found that knowledge and perception of risk was a significant determinant of greater compliance with recommended protective measures [23, 24]. However, in our study, being knowledgeable or perceiving risk did not always result in greater use of protective measures. Moreover, in general, the limited use of gloves and goggles while processing harvested birds was noted. These observations may be explained by the protection motivation theory which states that complying with a recommended protective health behavior is influenced by risk perception as well as efficacy variables, including response efficacy (i.e., whether the recommended measure is effective) and self-efficacy (i.e., whether the person is capable of performing the recommended measure) [57–59]. According to this theory, risk perception will generate a willingness to act, but efficacy variables will determine whether the resulting action is adaptive or maladaptive [57, 58]. In our study, those who perceived a risk may have doubted the effectiveness of recommended measures and/or had low self-efficacy owing to limited access to resources and ability to afford supplies required to implement the measures [60].

### Recommendations for influenza pandemic plans

These data support previous findings which suggest that bird hunting and processing activities may potentially expose individuals to avian influenza [5, 11–14]. Acknowledging the various benefits and cultural importance of subsistence harvesting [17, 20], while taking into account the increased hazard of potential AIV exposure in First Nations hunters, their inclusion as an avian influenza risk group with associated special considerations in pandemic plans seems warranted. The potential for a novel AIV to be introduced into an Aboriginal Canadian population is of great concern as they face many health disparities and are particularly susceptible to influenza and related complications [61]. Moreover, previous influenza pandemics have disproportionately impacted Aboriginal Canadians, especially those populations living in geographically remote communities, and reflected inadequacies in preparedness with regards to addressing their pre-existing inequalities and special needs during a pandemic [62–65].

Efforts should be directed towards improving education for First Nations hunters regarding avian influenza and the hazard posed by AIVs while harvesting wild birds. More specifically, our results indicated that educational endeavours should include information regarding the signs and symptoms of avian influenza, transmission dynamics, flyways of migrating birds, and recommended precautionary measures (Table 6). Accordingly, access to supplies required to comply with recommended protective measures, such as cleaning solutions and gloves, should be improved for First Nations subsistence hunters. Moreover, our findings suggested that detection of avian influenza in wild birds in nearby geographic areas would influence the participants’ harvesting behaviour. Given this, we recommend that a culturally-appropriate communication system be implemented to promptly inform subsistence hunters and other community members of the findings and any associated recommendations.

**Table 6:** **Recommended precautions for Canadian First Nations subsistence hunters to reduce exposure to avian influenza viruses while harvesting wild birds (adapted from [** [33]**])**

-	Do not touch or eat sick birds or birds that have died for unknown reasons
-	Avoid touching the blood, secretions, or dropping of wild game birds
-	Do not rub your eyes, touch your face, eat, drink or smoke when processing wild game birds
-	Keep young children away when processing wild game birds and discourage them from playing in areas that could be contaminated with wild bird droppings
-	When preparing game, wash knives, tools, work surfaces, and other equipment with soap and warm water followed by a household bleach solution (0.5% sodium hypochlorite)
-	Wear water-proof household gloves or disposable latex/plastic gloves when processing wild game birds
-	Wash gloves and hands (for at least 20 seconds) with soap and warm water immediately after you have finished processing game or cleaning equipment. If there is no water available, remove any dirt using a moist towlette, apply an alcohol based hand gel (between 60-90% alcohol) and wash your hands with soap and water as soon as it is possible
-	Change clothes after handling wild game birds and keep soiled clothing and shoes in a sealed plastic bag until they can be washed
-	When cooking birds, the inside temperature should reach 85°C for whole birds or 74°C for bird parts (no visible pink meat and juice runs clear)
-	Never keep wild birds in your home or as pets
-	Receive the annual influenza vaccine
-	If you become sick while handling birds or shortly afterwards, see your doctor and inform your doctor that you have been in close contact with wild birds.

### Study strengths and limitations

To our knowledge, this is the first study to examine the knowledge and risk perceptions of avian influenza among Canadian First Nations subsistence hunters. The censused approach taken to select participants and the high contact and cooperation rates strengthen the assertion that our findings are representative of the study community. Also, in accordance with a CBPR approach, the CBAG was involved throughout the entire research process, thereby ensuring that the study was conducted in a culturally-appropriate manner and that the knowledge generated was used to directly benefit the involved community.

Despite the novelty and significance of our findings, some limitations of our study must be highlighted when interpreting our results. First, the analysis was based on a cross-sectional survey of self-reported data which may limit drawing definitive conclusions about the observed relationships. The biases in recalling and reporting cannot be entirely ruled out; however, to help alleviate the potential for biased responses, participants were assured that their responses would remain anonymous. Also, it is not possible to discern whether those who did not return the survey or refused to participate were different in any way from those who did participate. However, there is no obvious reason to suspect that non-respondents and people who chose not to participate were any different from the respondents.

Future research should examine the prevalence of AIVs, particularly those strains that are currently of concern to humans (e.g., H5, H7), in birds from within the Mushkegowuk Territory that are typically harvested. Also, analyzing the sera for antibodies against AIV subtypes would be helpful to evaluate if previous AIV infections occurred in First Nation subsistence hunters. Moreover, conducting a quantitative exposure assessment would provide information to help characterize the study population’s exposure potential to AIVs. Lastly, previous research has noted that various barriers impede the effectiveness of implementing recommended pandemic mitigation measures [60]. Thus, future research should aim to understand if any barriers exist with regards to complying with recommended precautions to reduce exposure to AIVs while harvesting birds and if measures need to be adapted to be more context-specific and culturally-appropriate, while still maintaining the effectiveness of the measure.

## Conclusions

Our study aimed to gain an understanding of the bird harvesting practices and knowledge, risk perceptions, and attitudes regarding avian influenza among Canadian First Nations subsistence hunters and provide recommendations for pandemic plans. The findings herein indicated that First Nations subsistence hunters partook in some practices while harvesting wild birds that could potentially expose them to avian influenza, although appropriate levels of compliance with some protective measures were reported. More than half of the respondents were generally aware of avian influenza and almost one third perceived a risk of AIV infection while harvesting birds. Participants aware of avian influenza were more likely to perceive a risk of AIV infection while harvesting birds. Our results suggest that knowledge positively influenced the use of a recommended protective measure. Regarding attitudes about hunting influenza-infected birds, our results revealed that the frequency of First Nations hunters who would cease harvesting birds increased as AIV was detected in more nearby geographic areas.

Given that the potential exposure to AIVs while hunting is assumed to be low but the cultural importance of subsistence hunting high, our study indicated a need for more education about avian influenza and precautions First Nations hunters can take to reduce the possibility of AIV exposure while harvesting wild birds that are culturally-appropriate. We posit that First Nations hunters should be considered an avian influenza risk group and have associated special considerations included in pandemic plans.
